# Presence of Adipophilin-Positive Cancer-Associated Fibroblasts Is an Independent Poor Prognostic Indicator and Is Correlated with Immature-Type Desmoplastic Reaction in Patients with Colorectal Cancer

**DOI:** 10.3390/cancers17183006

**Published:** 2025-09-15

**Authors:** Toshinori Kobayashi, Mitsuaki Ishida, Hiroki Uehara, Shoichiro I, Norikazu Yamada, Yuto Igarashi, Chie Hagiwara, Yoshihiro Mori, Yoshinobu Hirose, Jun Watanabe

**Affiliations:** 1 Department of Colorectal Surgery, Kansai Medical University, 2-5-1, Shinmachi, Hirakata 573-1010, Japan; ueharah@hirakata.kmu.ac.jp (H.U.); ishoi@hirakata.kmu.ac.jp (S.I.); yamadan@hirakata.kmu.ac.jp (N.Y.); igarashy@hirakata.kmu.ac.jp (Y.I.); hagiwara.ch@ach.or.jp (C.H.); moriyo@hirakata.kmu.ac.jp (Y.M.); watanabj@hirakata.kmu.ac.jp (J.W.); 2 Department of Pathology, Osaka Medical and Pharmaceutical University, 2-7, Daigaku-machi, Takatsuki 569-8686, Japan; yoshinobu.hirose@ompu.ac.jp

**Keywords:** colorectal cancer, cancer-associated fibroblasts, desmoplastic reaction, lipid metabolism, lipid-laden cancer-associated fibroblasts

## Abstract

Background/Objectives: Immature-type (IM type) desmoplastic reaction (DR) is a poor prognostic indicator of CRC; however, the underlying mechanism has not yet been clarified. Recent studies have demonstrated that energy metabolism, including lipid metabolism, in cancer-associated fibroblasts (CAFs) can critically impact cancer cell growth. The prognostic significance of lipid-laden CAFs in CRC has not yet been elucidated. This study aimed to clarify whether the presence of lipid-laden CAFs correlates with the DR type and prognosis in CRC. Methods: We enrolled patients with pT3 or pT4 CRC who underwent surgical resection without neoadjuvant chemoradiotherapy. Immunohistochemical staining for adipophilin (ADP) was performed to determine the presence of lipid-laden CAFs. Results: The cohort included 70 patients (49 men and 21 women) with a median age of 74 years. ADP-positive lipid-laden CAFs were observed in 37 patients (53%). The presence of lipid-laden CAFs and ADP expression in carcinoma cells at the invasive front was significantly correlated with IM-type DR (*p* < 0.0001 and *p* = 0.0063, respectively). The presence of lipid-laden CAFs was an independent poor prognostic indicator based on multivariate analysis (hazard ratio: 3.65; 95% confidence interval: 1.082–13.038; *p* = 0.0368). Conclusions: These results suggest that the presence of lipid-laden CAFs enhance cancer progression, invasion, and metastasis, and that IM-type DR might be related to the lipid-rich tumor microenvironment.

## Simple Summary

Immature-type desmoplastic reaction (IM-type DR) is a histopathological indicator for poor prognosis in colorectal carcinoma (CRC); however, the underlying mechanism has not been clarified. Recent studies showed that lipid metabolism in cancer-associated fibroblasts (CAFs) can have critical impact on cancer cell growth. Our findings demonstrated that the presence of lipid-laden CAFs was an independent indicator of poor prognosis and was significantly correlated with presence of IM-type DR in CRC. Additionally, adipophilin expression in carcinoma cells at the invasive front was significantly correlated with both IM-type DR and the presence of lipid-laden CAFs. The presence of lipid-laden CAFs in CRC may enhance cancer progression and metastasis, and may link a lipid-rich tumor microenvironment.

## 1. Introduction

Colorectal cancer (CRC) is a common cause of cancer-related deaths worldwide, and its incidence is steadily increasing [1]. Therefore, establishing effective and useful prognostic indicators for patients with CRC is critical for prognostic stratification and developing treatment strategies in oncology [1]. The histopathological parameters of resected CRC specimens can provide pivotal information for prognosis. For example, histological differentiation grade, lymphovascular invasion, perineural invasion, and tumor budding (TB) have been recognized as established prognostic indicators [2,3,4,5,6]. Desmoplastic reaction (DR) is a tissue reaction that occurs around carcinoma cell nests to varying extents, such as presence of myxoid material or thick collagen bundles [7]. Types of DR were first reported as significant prognostic indicators in patients with pT3 or pT4 CRC by Ueno et al. [8], and subsequent articles have addressed their prognostic significance in other types of carcinomas [9,10]. The type of DR may reflect the tumor microenvironment (TME), which plays a pivotal role in tumor progression, invasion, and metastasis [7,8,9,10,11]. DR is classified into three categories: immature (IM), intermediate, and mature types, and IM-type DR, which is histopathologically characterized by the presence of myxoid material (amorphous mucoid material) at the invasive front of the tumor by hematoxylin and eosin staining, indicates a significantly worse prognosis in patients with CRC, followed by the intermediate and mature types, respectively (the detailed histopathological definition is described in the Histopathological analysis section) [7,8,12,13,14,15]. IM-type DR is significantly correlated with the presence of lymphovascular invasion, lymph node metastasis, and higher TB [7,8,12,13,14,15]. However, the mechanisms underlying its development and the relationship between the presence of IM-type DR and poor prognosis have not yet been elucidated, although the TME might influence the development of DR [7,12].

It has been demonstrated that energy metabolism markedly changes in carcinoma cells, and metabolic reprogramming is considered a hallmark of cancer [16]. The reprogramming of energy metabolism, including glucose, amino acids, and lipids, plays crucial roles in tumor initiation, progression, and metastasis [16,17,18,19,20], and lipid metabolism is usually upregulated in carcinoma cells [17,20]. Previous studies have extensively analyzed changes in energy metabolism in carcinoma cells [16,17,18,19,20]. Additionally, metabolic changes in the TME, such as in cancer-associated fibroblasts (CAFs) and inflammatory cells, have recently received much attention because it has been demonstrated that these changes could have a critical impact on cancer cell growth and metastasis via crosstalk between carcinoma cells and the TME [19,21,22]. Recent studies have demonstrated that the upregulation of lipid metabolism in CAFs plays a pivotal role in tumor growth and progression [23,24,25,26]. For example, Liu et al. demonstrated that lipid synthesis was upregulated in CAFs of oral squamous cell carcinoma, and these lipid metabolites were taken up by carcinoma cells, leading to tumor proliferation, migration, and invasion [23]. Niu et al. showed that pancreatic cancer cells facilitated lipid synthesis in CAFs, and these lipids were transferred to carcinoma cells, leading to the proliferation of cancer cells using a transgenic mouse model [24]. These CAFs are termed lipid-laden CAFs because lipid synthesis is upregulated, and lipids are stored in the cytoplasm [24]. Rich lipid storage can be visualized by immunohistochemical staining for adipophilin (ADP), a lipid-regulating protein that coats the surface of cytoplasmic lipid droplets [27]. Although upregulating lipid metabolism in CAFs in CRC has also been reported in a few articles [25,26], the relationship between the presence of lipid-laden CAFs, the type of DR, and prognosis has not yet been analyzed. This study aimed to examine the relationship between the presence of lipid-laden CAFs and the type of DR and their prognostic significance and to elucidate the correlation between the type of DR and prognosis.

## 2. Materials and Methods

### 2.1. Patient Selection

We enrolled consecutive patients with CRC who underwent surgical resection at the Department of Surgery of Kansai Medical University Hospital between January and December 2016. Patients with pT1 or pT2 disease were excluded since DR is defined as pT3 or pT4 [7,8,12,13,14,15]. Patients with lower or middle rectal cancers were excluded because neoadjuvant chemotherapy and/or radiation therapy were performed in most of these patients in our hospital. Patients who underwent neoadjuvant chemotherapy and/or radiation therapy were excluded. We set the observation window to 5 years according to the Japanese Society for Cancer of the Colon and Rectum guideline [28]. Moreover, no data regarding *KRAS* or *BRAF* mutation, and mismatch repair proteins status, were available.

The study cohort partially overlapped with that of our previous studies [2,29]. In one study, we showed that IM-type DR was significantly correlated with the presence of tumor deposits, which are discrete macroscopic or microscopic carcinoma nodules located in the extramural fatty tissue, and without lymph node structures [29]. In another study, we proposed a prognostic scoring system for patients with CRC, which was based on indicators reflecting the TME, including DR [2]. The results of this study did not overlap with those of these previous studies [2,29].

This retrospective study was conducted in accordance with the principles of the Declaration of Helsinki. The study protocol was approved by the Institutional Review Board of Kansai Medical University Hospital (Approval #2021-197). All data were anonymized. The Institutional Review Board waived the requirement for informed consent, because this was the retrospective study, and medical records and archived samples were used, and there was no risk to the participants. Additionally, this study did not include children. Information regarding this study was provided through the institutional website (https://www.kmu.ac.jp/hirakata/hospital/2671t800001356c-att/a1642567101597.pdf) (accessed on 27 July 2025).

### 2.2. Histopathological Analysis

Surgically resected specimens were fixed in 10% buffered neutral formalin, sectioned, and stained with hematoxylin and eosin. Two researchers (T.K. and M.I.) blinded to the clinical features independently evaluated the histopathological features of all tumor slides. The staging of patients with CRC is currently described in the American Joint Committee on Cancer Staging Manual, Eighth edition [30], which is based on the depth of tumor invasion (pT) and tumor lymph node and/or distant metastases (pN and M).

DR was classified into the IM, intermediate, and mature types according to the definition by Ueno et al. in patients with pT3 or 4 CRC [7,8,12,13,14,15]. Briefly, the IM type was histopathologically defined as the presence of myxoid stroma (amorphous mucoid material) greater than the microscopic field of ×400 at the invasive front of the carcinoma. The intermediate type was defined as the presence of keloid-like collagen (thick bundles of collagen accompanying hyalinization) without myxoid stroma, whereas the absence of myxoid stroma and keloid-like collagen was regarded as the mature type. In this study, DR was sub-classified into IM and non-IM (intermediate and mature) types.

TB was evaluated using the same method as previously reported [31]. Tumors with 0–4 buds (defined as the presence of a single tumor cell or a cluster of up to four tumor cells) at the invasive front were classified as TB1, 5–9 buds as TB2, and >10 buds as TB3.

### 2.3. Immunohistochemical Analysis

A whole section of the most morphologically representative carcinoma regions with DR, identified by hematoxylin and eosin-stained slides, was used for immunohistochemical staining of each tumor. Immunohistochemical staining was performed using an autostainer (Discovery ULTRA system; Roche Diagnostics, Basel, Switzerland) according to the manufacturer’s instructions. An OptiView DAB Universal Kit (760-700) was used for immunostaining. Dual immunohistochemical staining was performed using an autostainer (Leica BOND-MAX; Leica Biosystems GmbH, Nußloch, Germany) according to the manufacturer’s instructions. BOND Polymer Refine Detection Kit (DS9800; Leica) and BOND Polymer Refine Red Detection Kit (DS9390; Leica) were used. A primary mouse monoclonal antibody against ADP (AP125, ×100 dilution, Progen Biotechnik, Heidelberg, Germany) and a primary mouse monoclonal antibody against alpha-smooth muscle actin (alpha-SMA) (1A4, ×800 dilution, DAKO, Agilent Technologies, Inc., Santa Clara, CA, USA) were used. Human sebaceous glands and the muscularis propria of the colon were used as positive controls for ADP and alpha-SMA staining, respectively. Two researchers (T.K. and M.I.) blinded to the clinical or histopathological features independently evaluated the immunostaining results. When there were discrepancies of the results of the evaluation, a final decision was made by reassessment and agreement by two researchers.

First, we performed a single immunohistochemical staining for ADP in all CRC samples. ADP expression was evaluated in both the carcinoma cells and CAFs. If the tumors contained ADP-positive spindle cells around the carcinoma cell nests by single immunohistochemical staining for ADP, then dual immunohistochemical staining for ADP and alpha-SMA were performed to clarify whether these ADP-expressing spindle cells were CAFs or not. Both ADP and alpha-SMA were expressed in spindle cells, and these cells were recognized as lipid-laden CAFs. ADP-positivity in the carcinoma cells were evaluated by the morphological features as well as alpha-SMA negativity. ADP expression in both carcinoma cells and CAFs was identified as positive when carcinoma cells or CAFs exhibited granular and/or globular cytoplasmic expression, as previously reported [32,33,34,35,36]. When at least one ADP-expressing CAF was present, the tumor was classified as the presence of lipid-laden CAFs. We counted the percentage of ADP-expressing carcinoma cells (%). We also evaluated the presence of ADP-expressing carcinoma cells in the invasive front region of the carcinoma.

### 2.4. Statistical Analyses

JMP^®^ Student Edition version 18.2.1 (SAS Institute, Cary, NC, USA) was used for statistical analyses. Correlations between the two groups were analyzed using the chi-square test or Fisher’s exact test. Logistic regression analysis was performed to estimate the odds ratios (ORs) and 95% confidence intervals (CIs). Variables with a *p*-value < 0.20 in the univariate analysis were included to identify independent associations with histological features for multivariate logistic regression. To avoid overfitting in the multivariate Cox proportional hazards model, we selected up to three variables based on both clinical relevance and statistical significance in the univariate analysis, in accordance with the commonly recommended rule of at least 10 events per variable. Overall survival (OS) was analyzed using the Kaplan–Meier method, and survival differences between the groups were compared using the log-rank test. Statistical significance was set at *p* < 0.05.

## 3. Results

### 3.1. Clinicopathological Characteristics of the Study Cohort

**Figure 1:**
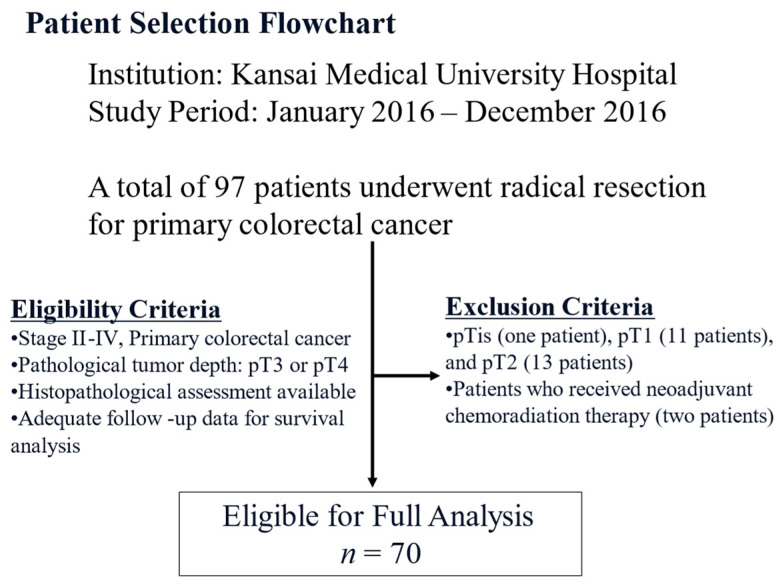
Flowchart of the patient selection process of the study cohort.

Table 1 summarizes the clinicopathological features of the study cohort. This study included 70 patients (29 [41%] women and 41 [59%] men). The median age at the time of surgery was 74 years (range: 47–89 years). The tumor locations were as follows: right-sided colon 40 (57%), left-sided colon 26 (37%), and upper rectum four patients (6%). Regarding the pathological T stage, 46 (66%) and 24 (34%) patients were classified as pT3 and pT4, respectively. Lymph node metastases were observed in 36 patients (51%). Thirty-two (46%), 25 (36%), and 13 (19%) patients were classified into pStages II, III, and IV, respectively. Figure 1 shows the flowchart of the patient selection process. The median observation period was 56.5 months (range: 0–77 months).

### 3.2. Histopathological Characteristics

**Figure 2:**
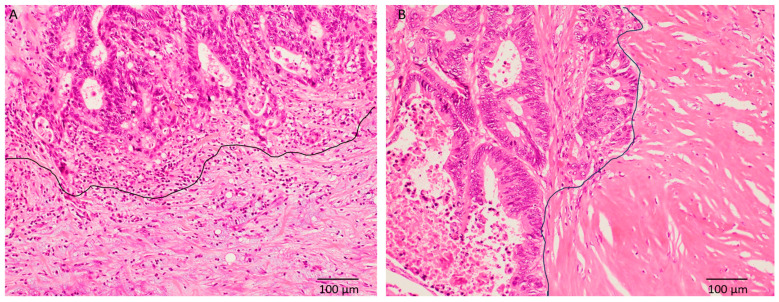
Typical histopathological features of desmoplastic reactions of colorectal carcinoma. (**A**) Immature type is characterized by the presence of myxoid material around the carcinoma cell nests at the invasive front (under area of the black line). (**B**) Presence of thick bundles of collagen without myxoid material (right area of the black line) is characteristic feature of intermediate type (hematoxylin and eosin, ×200).

Thirty-five (50%) patients each were classified into IM and non-IM type DR, respectively. The typical histopathological features of IM-type DR and non-IM type DR (intermediate type) are shown in Figure 2. IM-type DR was significantly correlated with pT4, the presence of lymph node metastasis, and higher pStage (*p* = 0.0118, 0.0001, and 0.0006, respectively) (Table 1).

### 3.3. Immunohistochemical Characteristics

**Figure 3:**
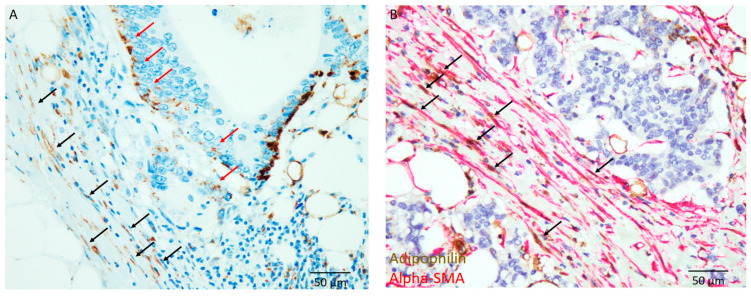
Immunohistochemical features. (**A**) Presence of adipophilin-expressing spindle cells around the carcinoma nests at the invasive front (black arrows). Adipophilin expression is also noted in carcinoma cells (red arrows) (×400). (**B**) Dual immunohistochemical staining demonstrates that both adipophilin (brown) and alpha-smooth muscle actin (alpha-SMA) (red) are expressed in spindle cells around the carcinoma nests (arrows). These spindle cells are considered lipid-laden cancer-associated fibroblasts (×400).

The typical features of ADP-positive lipid-laden CAFs at the invasive front are shown in Figure 3A. These ADP-positive lipid-laden spindle cells were also alpha-SMA-positive, according to dual immunohistochemical staining (Figure 3B). Therefore, these ADP-positive spindle cells at the invasive front were considered lipid-laden CAFs. Lipid-laden CAFs were observed in 37 patients (53%).

ADP expression was also observed in carcinoma cells. The median ratio of ADP-expressing carcinoma cells to total carcinoma cells was 10%; thus, we considered an ADP-high carcinoma if >10% of carcinoma cells showed positive immunoreactivity for ADP. Twenty-nine patients (41%) had ADP-high carcinomas (Table 1). ADP-high carcinomas were not significantly correlated with IM-type DR (*p* = 0.0894).

Additionally, ADP expression in carcinoma cells at the invasive front was observed in 51 patients (73%) (Figure 3A). ADP expression in carcinoma cells at the invasive front significantly correlated with IM-type DR (*p* = 0.0063).

### 3.4. Correlation Between Lipid-Laden CAFs and Clinicopathological Features

Table 2 summarizes the clinicopathological features of patients with or without lipid-laden CAFs. Lipid-laden CAFs were observed in 37 patients (53%) (28 and nine patients with IM-type and non-IM type DR, respectively), and the remaining 33 patients (47%) had no lipid-laden CAFs (seven and 26 patients with IM-type and non-IM type DR, respectively).

The presence of lipid-laden CAFs was significantly correlated with IM-type DR (*p* < 0.0001). It was also significantly correlated with the presence of lymph node metastasis, venous invasion, higher TB, and higher pStage (*p* = 0.0008, 0.0321, 0.0322, and 0.0035, respectively) but not with pT (*p* = 0.0946).

ADP-high carcinoma was not significantly correlated with the presence of lipid-laden CAFs (*p* = 0.873). However, ADP expression in carcinoma cells at the invasive front was significantly correlated with the presence of lipid-laden CAFs (*p* = 0.0014).

### 3.5. Prognostic Significance of the Type of DR and the Presence of Lipid-Laden CAFs

**Figure 4:**
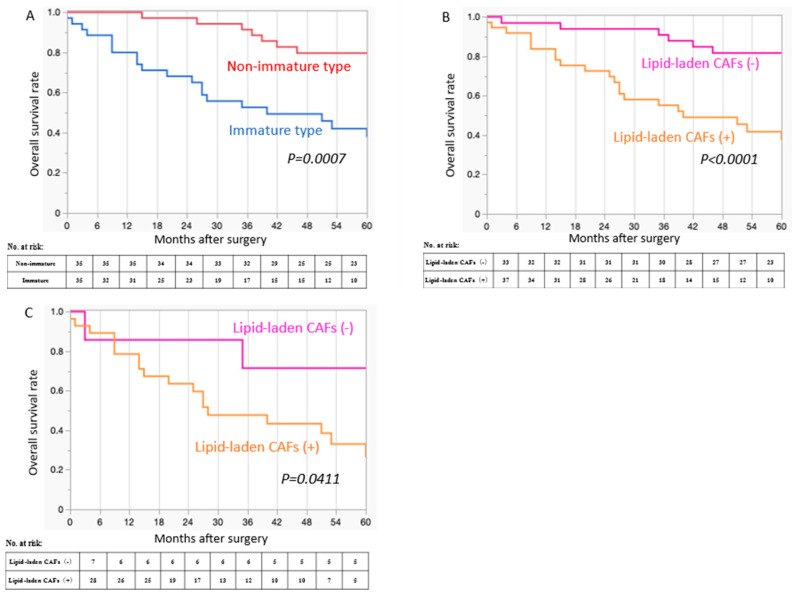
Overall survival curves. (**A**) The presence of immature-type desmoplastic reaction is a significant poor prognostic indicator compared to the non-immature type. (**B**) Presence of lipid-laden cancer-associated fibroblasts is a significant poor prognostic indicator in all patient cohorts. (**C**) Presence of lipid-laden cancer-associated fibroblasts is a significant poor prognostic indicator in patients with immature-type desmoplastic reaction.

Figure 4A shows the Kaplan–Meier curves of OS according to DR type (IM vs. non-IM). The presence of IM-type DR significantly correlated with poor OS (*p* = 0.0007). The median 5-year OS in patients with IM-type DR was 35 months (range 0–77 months), whereas that in patients with non-IM-type DR was 61 months (range 15–75 months).

The presence of lipid-laden CAFs was significantly correlated with poor OS in all patient cohorts (*p* < 0.0001) (Figure 4B). The median 5-year OS in patients with lipid-laden CAFs was 36 months (range 0–77 months), whereas that in patients without lipid-laden CAFs was 61 months (range 3–77 months). In patients with IM-type DR, the presence of lipid-laden CAFs significantly correlated with poor OS (*p* = 0.0411) (Figure 4C). The median 5-year OS of IM-type DR patients with lipid-laden CAFs was 27 months (range 0–77 months), while that of patients without lipid-laden CAFs was 71 months (range 3–77 months).

ADP-high carcinomas and ADP expression in carcinoma cells at the invasive front were not correlated with OS *p* = 0.9175 and 0.2001, respectively.

### 3.6. Cox Proportional Hazards Regression Analysis for OS

Table 3 shows the results of the univariate and multivariate Cox proportional hazards regression analyses for OS. The univariate analysis revealed that IM-type DR, the presence of lymph node metastasis, and presence of lipid-laden CAFs were significantly associated with OS (*p* = 0.0038, 0.0071, and 0.0005, respectively).

To avoid model overfitting due to the limited number of events, only three variables were selected for the multivariate Cox regression model, based on clinical relevance and statistical significance in the univariate analysis. The multivariate analysis revealed that the presence of lipid-laden CAFs was an independent predictor of poor OS (hazard ratio: 3.65; 95% CI: 1.082–13.038; *p* = 0.0368). IM-type DR and the presence of lymph node metastasis showed a trend toward poorer OS, although they were not statistically significant in the multivariate model (HR: 1.744, *p* = 0.387; and HR: 2.057, *p* = 0.172, respectively).

## 4. Discussion

This study clearly demonstrated that the presence of lipid-laden CAFs was an independent indicator of poor prognosis and was correlated with IM-type DR in CRC. Additionally, ADP expression in carcinoma cells at the invasive front was significantly correlated with both IM-type DR and the presence of lipid-laden CAFs. These results indicate that IM-type DR might be linked to a lipid-rich TME, and this environment might lead to a poor prognosis in patients with CRC. To our knowledge, this is the first study to address the prognostic significance of lipid-laden CAFs and their correlation with IM-type DR in CRC.

It is well recognized that reprogramming lipid metabolism is crucial for cancer cell growth and proliferation because lipids are essential sources of cell membranes and energy [17,20]. Over the past decades, changes in lipid metabolism in carcinoma cells have been extensively studied, and it has been revealed that *de novo* lipogenesis and lipid uptake are usually upregulated in carcinoma cells to meet their needs for rapid proliferation; reprogramming of lipid metabolism is recognized as one of the most remarkable changes in cancer metabolism [17,20]. Additionally, the significance of the TME in cancer growth, progression, and metastasis is well recognized, and changes in energy metabolism in the TME have received much attention [19,21,22]. Most studies on the metabolic reprogramming of CAFs have focused on changes in glycolysis and amino acids, such as glutamine [37]. CAFs reportedly produce large amounts of lactate, which are enhanced by aerobic glycolysis and decreased oxidative phosphorylation, as well as glutamine [38,39]. Upregulating lactate and glutamine can increase cancer cell proliferation and metastatic potential [37,38,39]. However, the significance of changes in lipid metabolism in CAFs has recently been highlighted in only a few studies [23,24,25,26]. Liu et al. showed that interleukin-8 secreted from oral squamous cell carcinoma cells activates lipid synthesis in CAFs via ATP citrate lyase, which catalyzes the conversion of citrate and coenzyme B to oxaloacetate and acetyl-CoA. These lipids are stored within the cytoplasm of CAFs, namely lipid-laden CAFs, and are subsequently released and absorbed by carcinoma cells, leading to cell proliferation, migration, and invasion [23]. Niu et al. demonstrated that bone morphogenic protein 2 produced by carcinoma cells induces lipid synthesis and storage in CAFs, and these lipid-laden CAFs, visualized by ADP staining, provide lipids to carcinoma cells, enhancing tumor progression in a mouse pancreatic cancer model [24]. Gong et al. reported that the combination of upregulation of fatty acid synthase (FASN), a key enzyme in fatty acid synthesis, in CAFs, and CD36, a fatty acid transporter, in carcinoma cells enhanced the migration ability of CRC [25]. Additionally, CRC cell-derived exosomes alter lipid metabolism in CAFs, establishing a premetastatic niche in the liver and promoting liver metastasis of CRC [22]. Accordingly, these results suggest that CAFs are a lipid source for carcinoma cells because lipid synthesis and storage are upregulated in CAFs, and these lipid metabolites are absorbed into carcinoma cells [23,24,25,26]. Metabolic reprogramming in both carcinoma cells and CAFs is thought to promote cancer proliferation, invasion, and metastasis. Therefore, the presence of lipid-laden CAFs in the TME may indicate upregulated lipid synthesis in CAFs, suggesting an increase in lipid metabolism in carcinoma cells. The results of this study showed that the presence of lipid-laden CAFs is an independent indicator of poor prognosis in patients with CRC and is significantly correlated with the presence of ADP-positive carcinoma cells at the invasive front. Accordingly, the upregulation of lipid metabolism at the invasive front, in collaboration with CFAs and carcinoma cells, might be suitable for cancer proliferation and progression, leading to a poor prognosis in patients with CRC. Moreover, Hsu et al. reported that oncogenic *KRAS* mutation transforms CAFs into lipid-laden CAFs, which promotes angiogenesis and progression of colon carcinoma, using mouse models [40]. Although *KRAS* mutation status was not available in the present cohort, presence of lipid-laden CAFs might be related to genetic change of CRC.

Previous studies have shown that ADP expression in carcinoma cells is a significantly poor prognostic indicator in some types of carcinomas, such as lung adenocarcinoma, pancreatic ductal adenocarcinoma, triple-negative breast cancer, salivary duct carcinoma, and metastatic CRC in the liver [32,33,34,35,36]. These results suggest that the upregulation of lipid metabolism in carcinoma cells leads to enhanced cancer proliferation, progression, and poor prognosis. ADP expression in carcinoma cells significantly correlates with lower FASN expression in triple-negative breast cancer and salivary duct carcinoma [35,41]. Additionally, FASN was reportedly upregulated in lipid-laden CAFs of pancreatic cancer in a mouse model [24]. Although ADP-high carcinomas and ADP expression in carcinoma cells at the invasive front did not correlate with survival in patients with CRC in this study, ADP expression in carcinoma cells at the invasive front was significantly correlated with the presence of lipid-laden CAFs. Accordingly, the upregulation of lipid synthesis in CAFs, downregulation of lipid synthesis, and upregulation of lipid intake in carcinoma cells may promote cancer cell proliferation and progression. Taken together, lipid-laden CAFs and carcinoma cells at the invasive front lead to the formation of a lipid-rich TME and the promotion of cancer cell proliferation and progression.

It is well established that IM-type DR is significantly correlated with poor prognosis in patients with CRC, which corresponds with our findings in this study. The DR types may represent the TME, including CAFs [7,8,12]. Significantly lower microvessel counts, lower inflammatory cell infiltrates, and higher tenascin-C and fibronectin expression have been observed in IM-type DR than in non-IM-type DR in CRC [8,12]. Fibronectin is a component of the extracellular matrix that plays a role in cell adhesion and migration and is involved in tissue remodeling and fibrosis. Therefore, myofibroblast activation in the TME may be correlated with the formation of IM-type DR and its aggressive clinical behaviour [12]. It has been speculated that the population and/or characteristics of CAFs differ among DR types in CRC [7]; however, the characterization of CAFs in IM-type DR has not been established. In this study, we clearly demonstrated that IM-type DR was significantly correlated with the presence of lipid-laden CAFs and ADP expression in carcinoma cells at the invasive front but not in ADP-high carcinomas. This result indicates that IM-type DR may be linked to a lipid-rich TME. Not all patients with IM-type DR had lipid-laden CAFs; however, the presence of lipid-laden CAFs in patients with IM-type DR was significantly correlated with poor OS. Therefore, although IM-type DR is not directly associated with the presence of lipid-laden CAFs, the presence of lipid-laden CAFs may also play an important role in cancer proliferation and progression, leading to poor OS.

This study had some limitations. First, it was conducted at a single institute with a relatively small sample size. Although this study included 70 patients with pT3 or pT4 CRC, a selection bias cannot be ruled out. Patients with rectal cancer who received neoadjuvant therapy were excluded because DR and the tumor microenvironment could be affected by neoadjuvant therapy. This may have limited the generalizability of the findings. Moreover, external validation using an independent cohort was not performed. Therefore, our findings should be interpreted as hypothesis-generating and require further validation in larger, multicenter cohorts. Second, this study clearly showed that both the presence of lipid-laden CAFs and ADP expression in carcinoma cells at the invasive front were significantly correlated with IM-type DR. Therefore, IM-type DR may be linked to the lipid-rich TME. However, our findings did not reveal the main player involved in lipid synthesis, carcinoma cells, or CAFs. Therefore, further studies are required to clarify the metabolic changes in both carcinoma cells and CAFs, as well as the molecular alterations in carcinoma cells. Third, a direct demonstration of the presence of lipids in the TME of IM-type DR was not performed because lipids are dissolved during the preparation of formalin-fixed and paraffin-embedded specimens. Fourth, the present cohort showed relatively high frequency of venous invasion. This might be related to the inclusion criteria of the present study. Specifically, all patients had pT3 or pT4 CRC, and 13 patients (19%) had stage IV disease. Therefore, the present cohort represented a relatively advanced population of CRC patients, which likely contributed to the high incidence of venous invasion. Moreover, no genetic data of carcinoma cells, as well as immunological markers, were available in the present cohort. Presence of lipid-laden CAFs might be related to genetic changes because KRAS mutation status can transform CAFs into lipid-laden CAFs in mouse models [40]. Therefore, further studies are needed to clarify the genetic alterations in patients with lipid-laden CAFs.

## 5. Conclusions

This study clearly demonstrated that the presence of lipid-laden CAFs and ADP expression in carcinoma cells at the invasive front were significantly correlated with IM-type DR. The presence of lipid-laden CAFs may be linked to a lipid-rich TME, which might enhance cancer cell proliferation, invasion, and metastasis. Further studies are needed to clarify the mechanism underlying IM-type DR with respect to metabolic changes in both lipid-laden CAFs and carcinoma cells.

## Figures and Tables

**Table 1:** Clinicopathological features between immature and non-immature-type desmoplastic reactions.

Variables	Total Number (%)	Immature, *n* = 35 (%)	Non-Immature, *n* = 35 (%)	*p*-Value
Median age, years (range)	74 (47–89)	73 (58–86)	74 (47–89)	0.976
Sex				
Male	41 (59)	20 (57)	21 (60)	0.808
Female	29 (41)	15 (43)	14 (40)	
Location				
Right-side	40 (57)	16 (46)	24 (68)	0.136
Left-side	26 (37)	16 (46)	10 (29)	
Rectum	4 (6)	3 (8)	1 (3)	
pT				
pT3	46 (66)	18 (51)	28 (80)	0.0118
pT4	24 (34)	17 (49)	7 (20)	
pN				
Negative	34 (49)	9 (26)	25 (71)	0.0001
Positive	36 (51)	26 (74)	10 (29)	
pStage				
II	32 (46)	8 (23)	24 (69)	0.0006
III	25 (36)	17 (48)	8 (23)	
IV	13 (18)	10 (29)	3 (8)	
Tumour differentiation				
Low-grade	66 (94)	33 (94)	33 (94)	
High-grade	4 (6)	2 (6)	2 (6)	0.99
Tumour budding				
TB1	21 (30)	9 (25)	12 (34)	
TB2+3	49 (70)	26 (75)	23 (66)	0.433
Lymphatic invasion				
Negative	24 (34)	12 (34)	12 (34)	
Positive	46 (66)	23 (66)	23 (66)	0.99
Venous invasion				
Negative	7 (10)	2 (5)	5 (14)	0.232
Positive	63 (90)	33 (95)	30 (86)	
Lipid-laden cancer-associated fibroblasts				
Absent	33 (47)	7 (20)	26 (74)	<0.0001
Present	37 (53)	28 (80)	9 (26)	
Adipophilin expression in total carcinoma cells (%) median (range)	10 (0–90)	15 (0–90)	5 (0–90)	0.1207
Adipophilin expression in carcinoma cells				
Low (<10%)	41 (58)	17 (49)	24 (69)	0.0894
High (≧10%)	29 (42)	18 (51)	11 (31)	
Adipophilin expression in carcinoma cells at the invasive front				
Absent	19 (27)	4 (11)	15 (43)	0.0063
Present	51 (73)	31 (89)	20 (57)	

**Table 2:** Correlation between clinicopathological features and lipid-laden cancer-associated fibroblasts.

	Lipid-Laden Cancer-Associated Fibroblasts		
Variables	Present, *n* = 37 (%)	Absent, *n* = 33 (%)	*p*-Value
Median age, years (range)	75 (58–89)	71 (47–85)	0.145
Sex			
Male	21 (57)	20 (60)	0.744
Female	16 (43)	13 (40)	
Location			
Right-side	22 (60)	18 (55)	0.514
Left-side	12 (32)	14 (42)	
Rectum	3 (8)	1 (3)	
pT			
pT3	21 (56)	25 (76)	0.0946
pT4	16 (43)	8 (24)	
pN			
Negative	11 (30)	23 (70)	0.0008
Positive	26 (70)	10 (30)	
pStage			
II	10 (27)	22 (66)	0.0035
III	17 (46)	8 (24)	
IV	10 (27)	3 (10)	
Tumor differentiation			
Low-grade	35 (96)	31 (94)	0.906
High-grade	2 (4)	2 (6)	
Tumor budding			
TB1	7 (19)	14 (42)	
TB2+3	30 (81)	19 (58)	0.0322
Lymphatic invasion			
Negative	12 (32)	12 (36)	0.7294
Positive	25 (68)	21 (64)	
Venous invasion			
Negative	1 (3)	6 (18)	0.0321
Positive	36 (97)	27 (82)	
Desmoplastic reaction			
Immature type	28 (76)	7 (21)	<0.0001
Non-immature type	9 (24)	26 (79)	
Adipophilin expression in carcinoma cells			
Low (<10%)	22 (59)	19 (58)	0.873
High (≧10%)	15 (41)	14 (42)	
Adipophilin expression in carcinoma cells at the invasive front			
Absent	4 (11)	15 (45)	0.0014
Present	33 (89)	18 (55)	

**Table 3:** Univariate and multivariate analyses of prognostic factors.

Variables	Dead Patients *n* = 30 (%)	Alive Patients *n* = 40 (%)	Univariate *p*-Value	Multivariate *p*-Value	HR	95% CI
pT4	14 (47)	10 (25)	0.0588	-	-	-
Immature-type desmoplastic reaction	21 (70)	14 (35)	0.0038	0.387	1.744	0.482–6.094
Presence of lymph node metastasis	21 (70)	15 (38)	0.0071	0.172	2.057	0.631–6.671
Presence of lymphatic invasion	21 (70)	25 (63)	0.513	-	-	-
Presence of vascular invasion	29 (97)	34 (85)	0.107	-	-	-
Tumor differentiation (high-grade)	3 (10)	1 (2.5)	0.181	-	-	-
Tumor budding (TB) 2+3	23 (77)	26 (65)	0.291	-	-	-
Adipophilin expression in carcinoma cells (more than 10%)	14 (47)	15 (38)	0.441	-	-	-
Presence of adipophilin expression in carcinoma cells at the invasive front	24 (80)	27 (68)	0.244	-	-	-
Presence of lipid-laden cancer-associated fibroblasts	23 (77)	14 (35)	0.0005	0.0368	3.65	1.082–13.038

HR, hazard ratio; CI, confidence interval.

## Data Availability

Due to the nature of this research, participants in this study could not be contacted about whether the findings could be shared publicity. Thus, supporting data are not available. The datasets generated and analysed during the current study are not publicly available due to the nature of the research but are available from the corresponding author on reasonable request.

## Abbreviations

The following abbreviations are used in this manuscript:

ADP	Adipophilin
alpha-SMA	alpha-smooth muscle actin
CAF	Cancer-associated fibroblast
CRC	Colorectal cancer
DR	Desmoplastic reaction
FASN	Fatty acid synthase
IM type DR	Immature-type desmoplastic reaction
OS	Overall survival
TB	Tumor budding
TME	Tumor microenvironment
